# Economic inequality and crime across cities in India: Evidence using nighttime lights data

**DOI:** 10.1371/journal.pone.0324937

**Published:** 2025-08-11

**Authors:** Christopher Kuruvilla Mathen, Siddhartha Chattopadhyay, Sohini Sahu, Abhijit Mukherjee

**Affiliations:** 1 Department of Humanities and Social Sciences, Indian Institute of Technology Kharagpur, Kharagpur, West Bengal, India; 2 Department of Economic Sciences, Indian Institute of Technology Kanpur, Kanpur, Uttar Pradesh, India; 3 Department of Geology and Geophysics, Indian Institute of Technology Kharagpur, Kharagpur, West Bengal, India; Northeastern University, UNITED STATES OF AMERICA

## Abstract

This study investigates the relationship between economic inequality and crime rates across 49 Indian cities from 2016 to 2021. Given the limited availability of traditional economic indicators at the city level in India, we introduce a novel methodological approach using nighttime lights collected by the Suomi National Polar-orbiting Partnership (SNPP) Visible Infrared Imaging Radiometer Suite (VIIRS) satellites and its inequality as an alternative indicator for economic activity and economic inequality. Employing a dynamic panel data model, we demonstrate that inequality based on nighttime lights has a statistically significant positive impact on crime rates. Our findings reveal that a 1% increase in light-based inequality corresponds to a 0.5% increase in total crime rate. This relationship remains robust across multiple crime categories, including violent crime, minor property crime, and property crime. Furthermore, we observe that conviction rates have a significant negative impact on total crime rate, suggesting the deterrent effect of effective law enforcement. These results contribute to the growing literature on urban crime dynamics and offer insights on the use of remotely sensed nighttime lights for studying economic disparities in regions where conventional economic data may be limited.

## 1. Introduction

The rapid urbanization in developing countries with uneven development has led to a rise in criminal activities in cities. Urban regions are prone to higher incidences of crime due to the higher returns from illegal activities and higher economic opportunities [1]. In sociological paradigm, *strain theory* states that regions with high inequality are characterised by a higher incidence of crime because a section of society is deprived of their legitimate needs [2]. Subsequently, the *envy effect* of the residents in cities with high inequality results in higher crime rates [3]. Several studies have examined the detrimental effect of inequality in rising crime rate at the cross-country and at the subnational level [4–8]. Following the seminal study by [9], several studies have also considered crime as a rational activity where an individual evaluates the cost and benefits associated with committing the crime before engaging in criminal activities [10–12]. Consequently, a higher conviction rate reduces crime rates as the increased probability of conviction raises the cost of engaging in illegal activities [13,14].

India is characterised by uneven development with disparity in household amenities and wealth across regions [15]. National Crime Records Bureau (shortly known as NCRB) is the official agency to provide crime statistics in India. NCRB provides crime records for different types of crime aggregated at the city, district, state and national level. The Crime in India report, annual publication of NCRB, discusses in detail the various aspects of crime at different subnational levels in India. Despite the availability of city-level crime rates in India, only few studies have analysed crime rate across cities in India but mostly rely on descriptive statistics or qualitative techniques [16,17]. The limitation in employing quantitative techniques is because the socio-economic variables are unavailable across cities in India. To overcome the unavailability of economic indicators on economic activity and inequality at the city level in India, we use nighttime lights obtained from Suomi National Polar-orbiting Partnership (SNPP) Visible Infrared Imaging Radiometer Suite (VIIRS) satellites and its inequality measured using the Gini coefficient as a proxy measure of economic activity and economic inequality, respectively, to examine the crime rate at the city level. The seminal papers by [18,19] have led to the use of nighttime lights and its inequality as a proxy measure of economic activity and economic inequality in numerous studies [20–28].

Our objective is to examine how inequality affects crime rate across the cities in India, along with the deterrent effect of conviction rate on crime rate. Using dynamic panel data model across 49 cities in India, our findings suggest that cities with higher inequality have higher crime rate in India. However, the impact varies across different types of crime with nighttime light inequality having higher impact on violent crime, and relatively lower impact on minor property crime and property crime. Therefore, a uniform spatial development at the city level can reduce crime rate across cities in India. Further, we find that rise in conviction rate has a deterrent effect on crime rate across cities in India.

## 2. Materials and methods

### 2.1. Data

**Fig 1:**
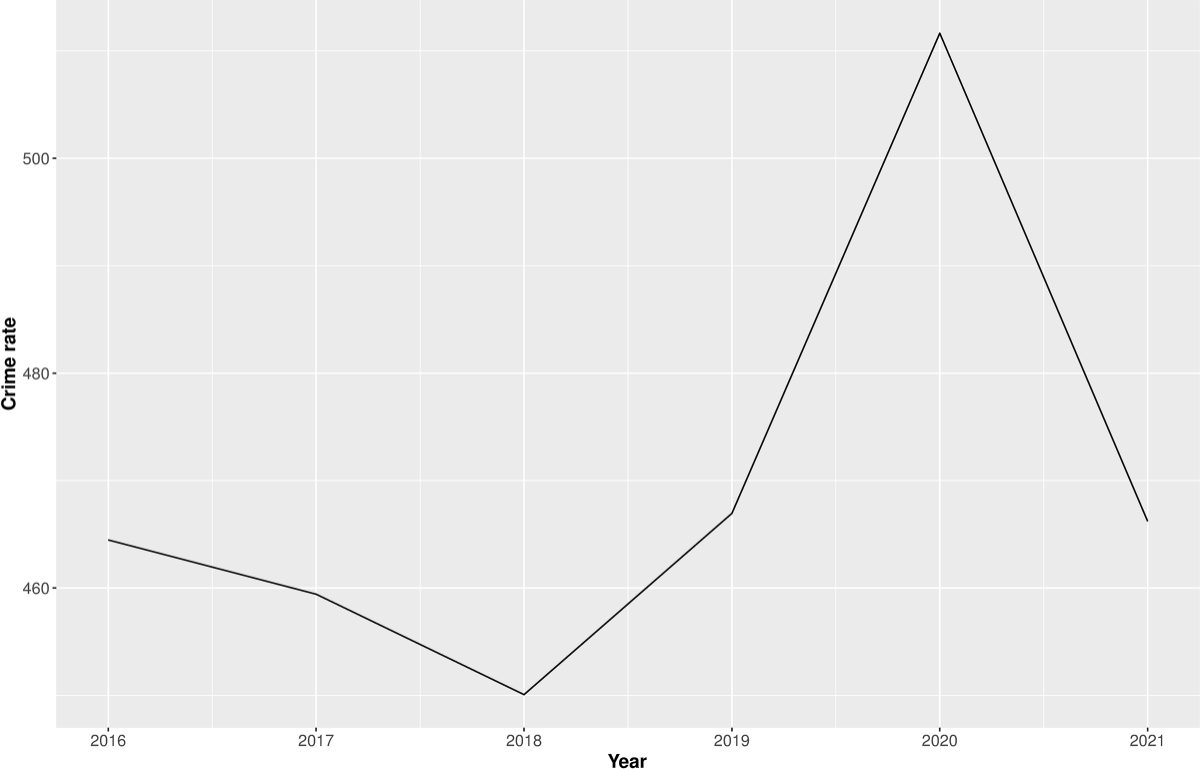
Total crime rate (city-level) in India from 2016-2021.

The incidence of crime and total convicted cases are obtained from the National Crime Records Bureau (NCRB). The total crime rate, incidence of total crime per 100,000 persons, across cities in India has increased from 464 in 2016 to 512 in 2020, which then declined to 466 in 2021. Fig 1 shows the total crime rate, i.e., the incidence of total crime per 100,000 persons, in India from 2016 till 2021.

**Fig 2:**
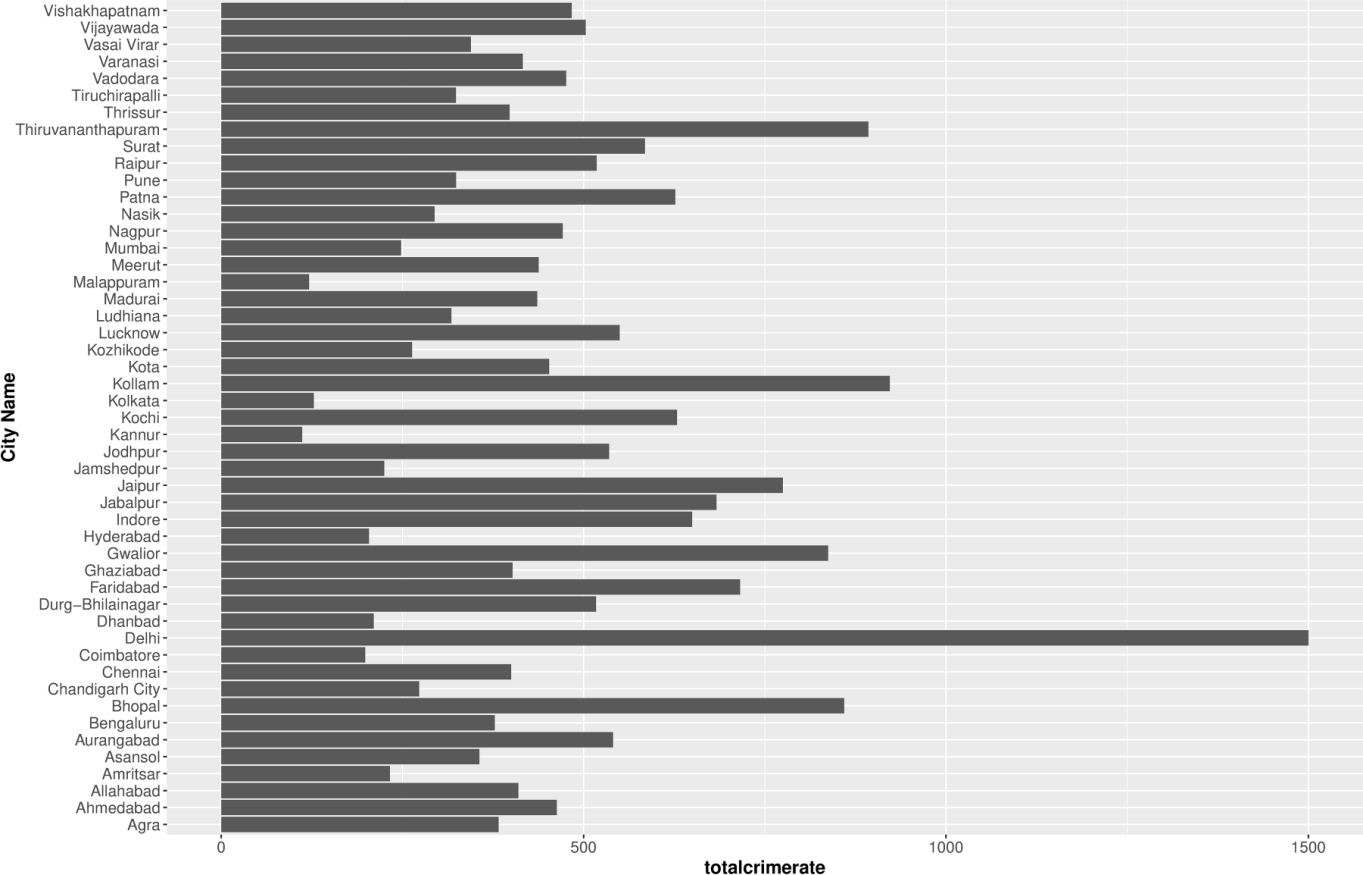
Total crime rate across cities in India.

We have compiled information for 49 cities in India during the period between 2016–2021 available at an annual interval. NCRB provides crime data for 53 cities in India which has population greater than 1 million as per census 2011. The major omission due to the population criterion is the city level crime data for the north eastern states in India. Although the inclusion of the cities in north eastern states, with its varied socioeconomic conditions, could provide further insights into the impact of inequality on crime, due to the unavailability of the data, we are unable to account for it. Further, the observations for Kanpur, Srinagar and Ranchi were removed due to the unavailability of city level boundaries, whereas Rajkot was removed due to data quality issues resulting in observation for 49 cities across 2016–2021. There were zero convicted cases for Agra for the years 2018 and 2021, and Ghaziabad for the year 2019 which results in 291 observations. Fig 2 shows that the total crime rate is spatially heterogenous across 49 cities in India, with Delhi, a union territory having the highest total crime rate (1500), followed by Kollam (922) and Thiruvananthapuram (892), both in the state of Kerala. The cities with the lowest total crime rate are Kannur (111) and Malappuram (121), both in the state of Kerala, followed by Kolkata (127), in the state of West Bengal. The conviction rate or the probability of conviction is computed by dividing the total convicted cases by the incidence of total crime.

**Fig 3:**
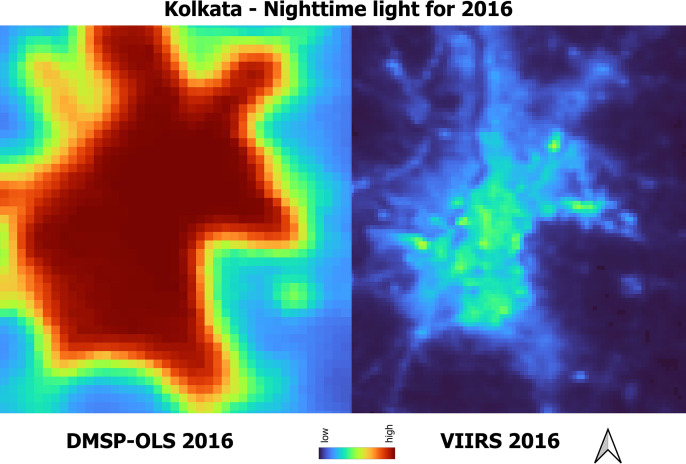
Nighttime lights of Kolkata city in India.

**Fig 4:**
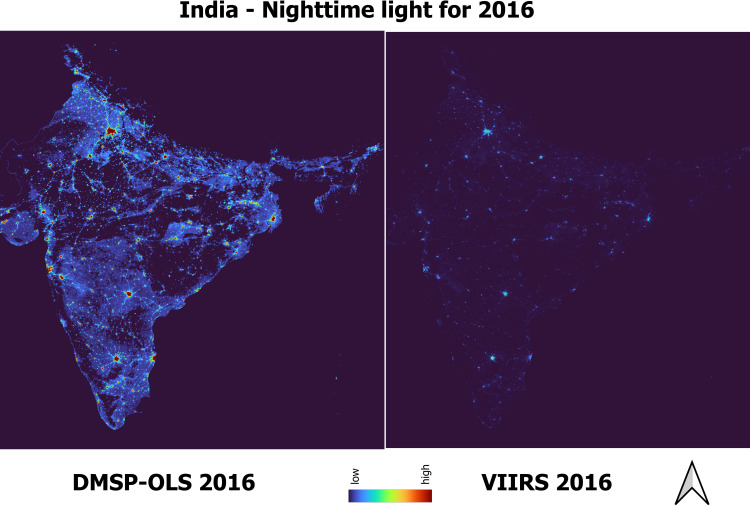
Nighttime lights of India.

The boundaries for the city are extracted from OpenStreetMap. We use Tehsil/township/district boundaries when the shapefiles for the city boundaries are not available. The city boundaries are provided in the GitHub repository (see https://github.com/chrismathen/NTLineq). Majorly, two satellite sources collects nighttime lights data – i) the Defense Meteorological Satellite Program Operational Line-Scan System (DMSP-OLS), available at a resolution of 2.7 km, from 1992–2013 [29], and (ii) the Suomi National Polar-orbiting Partnership (SNPP) Visible Infrared Imaging Radiometer Suite (VIIRS), available at a resolution of around 500m [30], from 2012 onwards. Although the nighttime lights data collected by DMSP-OLS satellite are extended beyond 2013 [29], due to the outdated technology used in DMSP-OLS satellite, measurements errors exist in nighttime lights data collected by DMSP-OLS satellite [31–36]. The measurement errors in nighttime lights data collected by DMSP-OLS satellites due to i)overglow or blurring, (ii) geolocation errors, (iii)time series errors, (iv) lack of calibration across satellites, and (v) top coding issues due to limited dynamic range are discussed by [37,38]. Due to the inherent measurement errors associated with nighttime lights obtained from DMSP-OLS satellites, the inequality computed on nighttime lights collected by DMSP-OLS satellites would be understated [34,36]. Therefore, we use nighttime lights collected by VIIRS satellites as a proxy measure of economic activity and its inequality in this study. Fig 3 shows the nighttime lights collected by DMSP-OLS and VIIRS satellites of Kolkata city for the year 2016. Fig 3 shows that the regions within and nearer to Kolkata are almost as lit for nighttime lights collected by DMSP-OLS satellites which results in understatement of inequality based on nighttime lights. Fig 4 shows the nighttime lights collected by DMSP-OLS and VIIRS satellites for India (). The understatement of inequality based on nighttime lights collected by DMSP-OLS satellites due to blurring and top-coding issue for India is discussed by [36].

**Fig 5:**
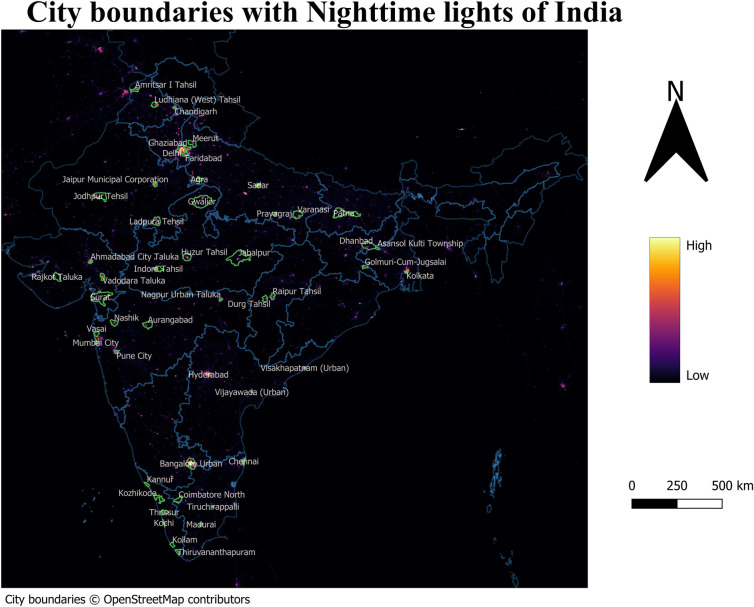
City boundaries and Nighttime lights of India obtained from VIIRS satellites.

Therefore, we extract annual nighttime lights for the cities from nighttime lights (see Fig 5) obtained by Suomi National Polar-orbiting Partnership (SNPP) Visible Infrared Imaging Radiometer Suite (VIIRS) satellites version 2.1 provided by Earth Observation Group, Payne Institute for Public Policy [30]  . Population density is extracted from LandScan images from 2016 to 2021 [39], and are available at an annual interval.

While the spatial resolution of VIIRS is around 500m, the spatial resolution of LandScan images are approximately around 1 km [39]. Therefore, we generate 1 km × 1 km rectangle grid-cell based on NSIDC Equal Area Scalable Earth (EASE) Grid – 2.0 global Coordinate Reference System (CRS), and intersect it with the city level boundaries to obtain 1 km × 1 km grid-cell (or spatial polygon) within each 49 cities in India. Although using smaller and finer grid-cells can improve the accuracy by capturing local variations in nighttime lights, especially in dense urban areas, the spatial resolution of LandScan images are approximately around 1 km [39], which limits our size of grid-cell to 1 km × 1 km in this study. We export the shapefile to WGS84 4326 Coordinate Reference System (CRS) to be consistent with the CRS of the raster file. Further, based on multiprocessing algorithm developed in python (version 3.11.0), we extract the nighttime lights and population for the 1 km × 1 km spatial polygon for each year from 2016 to 2021. Inequality based on nighttime lights extracted for 1 km × 1 km grid-cell within each 49 cities in India is computed using equation (1), following [26]. We use population-weighted Gini coefficient to measure inequality:


$$\begin{array}{c} G_{w}=\frac{\sum_{i=1}^{m}\sum_{k=1}^{m}p_{i}p_{k}|x_{i}-x_{k}|}{2{\overset{―}{x}}_{w}}\; \end{array}$$ (1)

where x represents percapita nighttime lights of $i^{th}\;and\;k^{th}\;$1km × 1 km grid-cell within the city, p denotes the population density for 1 km × 1 km grid-cell within the city, and ${\overset{―}{x}}_{w}$ is the weighted mean of percapita nighttime lights emission. We have used R software (version 4.3.1) to compute the inequality based on nighttime lights. The shapefile and the code used to extract nighttime lights and compute inequality are available in our GitHub repository (see https://github.com/chrismathen/NTLineq).

### 2.2. Empirical model

We specify crime rate as a function of economic factors and legal deterrence factors in equation (2). To capture the nonlinearity of economic activity proxied using nighttime lights on crime rate, we use nighttime lights squared in the regression specification [40]. We have also estimated equation (2) assuming linear relationship between economic activity proxied using nighttime lights and crime rate. The results are provided in S1 Tables 2 and 3 in S1 Appendix.


$$\begin{array}{c} {\ln(Crime)}_{i,t}=\alpha_{i}+\eta_{t}+\beta_{1}{\ln(Gini)}_{i,t}+\beta_{2}{\ln(ntl)}_{i,t}+\beta_{3}{\ln(ntl)}_{i,t}^{2}+\beta_{4}{\ln(convictionrate)}_{i,t}+\epsilon_{i,t},\; \end{array}$$ (2)

where $\begin{array}{c} {\ln(Crime)}_{i,t} \end{array}$ is the log transformed incidence of crime for $i^{th}$ city in time t. $\begin{array}{c} {\ln(ntl)}_{i,t} \end{array}$ is the economic activity for $i^{th}$ city in time t proxied using nighttime lights and $\begin{array}{c} {\ln(ntl)}_{i,t}^{2} \end{array}$ is the squared term. $\begin{array}{c} {\ln(convictionrate)}_{i,t} \end{array}$ is the probability of conviction, i.e., number of cases convicted divided by the total number of cases multiplied by 100. $\alpha_{i}\;$ controls for the time-invariant omitted variables at city level and $\eta_{t}$ controls for the time specific shocks commonly affecting all cities.

However, following the literature [40–42], we account for the persistence or inertial effect of crime and modify equation (2) into a dynamic panel data model by incorporating the crime rate lagged by a time period in equation (3).


$$\begin{array}{c} {\ln(Crime)}_{i,t}=\alpha_{i}+\eta_{t}+\lambda {\ln(Crime)}_{i,t-1}+\beta_{1}{\ln(Gini)}_{i,t}+\beta_{2}{\ln(ntl)}_{i,t}+\beta_{3}{\ln(ntl)}_{i,t}^{2}+\;\beta_{4}{\ln(convictionrate)}_{i,t}+\epsilon_{i,t}\; \end{array}$$ (3)

Equation (3), when estimated with a fixed effect estimator leads to Nickell bias [43] i.e., C$ov({Crime}_{i,t-1}-\overset{―}{{Crime}_{i,t-1,}},\epsilon_{s,t}-\overset{―}{\epsilon_{s}})\neq 0$, where $\overset{―}{{Crime}_{s,t-1,}}=\frac{1}{T-1}\sum_{t=2}^{T}{Crime}_{s,t-1}$. Therefore, we address the endogeneity concern by estimating equation (2) using generalized method of moments (GMM) estimation [41,44]. We use system GMM specification as it is more efficient than difference GMM estimation. System GMM relies on the additional assumption that the first differences of the instrumental variables are orthogonal with the fixed effects [45–47]. The summary statistics for the variables are provided in the S1 Table 1 in S1 Appendix.

## 3. Empirical results

The results for the effect of inequality based on nighttime lights on the total crime rate across cities in India are provided in Table 1. Column (1) in Table 1 presents the results for the fixed effects estimation based on equation (2), column (2) presents the estimation results for the dynamic panel data model based on equation (3), and lastly, column (3) presents the results for the system GMM estimation based on equation (3). Across all the columns, the coefficient for inequality based on nighttime lights is positive and statistically significant. We explain Column (3) in detail as it is our benchmark result. Column (3) in Table 1 shows that inequality based on nighttime lights has a positive and statistically significant impact on total crime rate across Indian cities. We find that a 1% increase in inequality based on nighttime lights leads to a 0.5% increase in total crime rate across 49 Indian cities. As expected, based on the literature review [9,10], a higher conviction rate has a negative and statistically significant impact on the total crime rate across cities in India across all columns. Column (3) in Table 1 shows that a 1% increase in the conviction rate leads to a 0.12% decline in the total crime rate across cities in India. However, we do not find evidence for a statistically significant relationship between nighttime lights and population density with total crime rate. The results from Table 1 shows that the sign and the statistical significance of the coefficients for inequality based on nighttime lights and conviction rate are robust across all the columns, underpinning the significance of inequality and conviction rate in combating total crime rate.

**Table 1:** Impact of inequality on Total crime rate across Indian cities.

	Dependent variable: log (crime rate)		
	(1)	(2)	(3)
	FE	FE with lag	SystemGMM
L.log (crime rate)		0.125	0.233^**^
		(0.0906)	(0.0915)
log (Nighttime light inequality)	0.695^**^	0.749^*^	0.482^*^
	(0.273)	(0.404)	(0.290)
log (conviction rate)	−0.0725^*^	−0.102^**^	−0.123^***^
	(0.0374)	(0.0421)	(0.0265)
log (nighttime lights)	−0.369	0.276	0.212
	(0.717)	(0.912)	(0.996)
log (nighttime lights)^2	0.0162	−0.0102	0.0000550
	(0.0446)	(0.0582)	(0.0541)
log (population density)	0.399	0.191	−0.0747
	(0.278)	(0.278)	(0.174)
Constant	5.065	2.085	2.813
	(3.834)	(4.120)	(4.235)
Observations	291	242	242
No: of City	49	49	49
City fixed effect	Yes	Yes	Yes
Year fixed effect	Yes	Yes	Yes
Within R-squared	0.125	0.142	
Number of instruments			24
AR (1) test (p-value)			0.0018
AR (2) test (p-value)			0.7169
Sargan test (p-value)			0.0928

L.log (crime rate) is the crime rate lagged by one year. Crime rate is defined as the total crime recorded per 100,000 persons. Inequality based on nighttime lights is the Gini coefficient of nighttime lights used as a proxy measure of inequality. All variables in the analysis have been log-transformed. Statistical significance is denoted as follows:

*** ,

** ,

* statistically significant at the 1% level, 5% level, and 10% level, respectively. Standard errors clustered at the city level are provided in parenthesis. The p-values for the AR (1), AR (2), and Sargan overidentification tests are also reported.

Further, the study time period, 2016−2021, also include the years for which COVID-19 pandemic may have an impact on the total crime rate across cities in India. COVID-19 lockdown in India was imposed on 25 March, 2020, and was only gradually lifted over the months starting from June, 2020. Therefore, we specify COVID-19 dummy for the years 2020 and 2021 as 1, and 0 otherwise to incorporate the impact of COVID-19 pandemic on total crime rate across 49 Indian cities. Table 2 provides the results incorporating the impact of COVID-19 pandemic on the incidence of total crime across Indian cities. Column (1) in Table 2 presents the results for the fixed effects estimation, column (2) shows the results for the dynamic panel data model, and lastly, column (3) shows the results for the system generalized method of moments estimation. In similar vein to the results in Table 1, we find inequality to have a statistically significant positive impact on total crime rate across Indian cities. The coefficient for nighttime light inequality in column (3) is 0.757 and is statistically significant at 10% level of significance. Table 2 also provides robust evidence on the negative impact of conviction rate on total crime rate across Indian cities. Although, the coefficient for COVID-19 dummy is negative in column (3) in Table 2, we do not find COVID-19 pandemic to have a statistically significant impact on total crime rate across cities in India.

**Table 2:** Impact of inequality on Total crime rate across Indian cities controlling for the impact of COVID-19 pandemic.

	Dependent variable: log (crime rate)		
	(1)	(2)	(3)
	FE	FE with lag	SystemGMM
L.log (crime rate)		0.114	0.346^*^
		(0.0959)	(0.187)
log (Nighttime light inequality)	0.657^**^	0.708^*^	0.757^*^
	(0.266)	(0.395)	(0.458)
log (conviction rate)	−0.0802^**^	−0.111^**^	−0.157^*^
	(0.0384)	(0.0435)	(0.0881)
log (nighttime lights)	−0.332	0.328	0.207
	(0.712)	(0.902)	(1.423)
log (nighttime lights)^2	0.0122	−0.0147	0.000617
	(0.0442)	(0.0566)	(0.0772)
log (population density)	0.370	0.177	−0.287
	(0.259)	(0.264)	(0.226)
Covid-19 dummy	0.00592	−0.0214	−0.0369
	(0.0544)	(0.0596)	(0.0794)
Constant	5.231	2.109	3.901
	(3.736)	(4.149)	(5.899)
Observations	291	242	242
No: of City	49	49	49
City fixed effect	Yes	Yes	Yes
Within R-squared	0.119	0.135	
Number of instruments			40
AR (1) test (p-value)			0.0013
AR (2) test (p-value)			0.5895
Sargan test (p-value)			0.305

L.log (crime rate) is the crime rate lagged by one year. Crime rate is defined as the total crime recorded per 100,000 persons. Inequality based on nighttime lights is the Gini coefficient of nighttime lights used as a proxy measure of inequality. All variables in the analysis have been log-transformed. Statistical significance is denoted as follows:

*** ,

** ,

* statistically significant at the 1% level, 5% level, and 10% level, respectively. Standard errors clustered at the city level are provided in parenthesis. The p-values for the AR (1), AR (2), and Sargan overidentification tests are also reported. Covid-19 dummy for the year 2020 and 2021 are marked as 1, otherwise 0.

Further, following the literature [13,41,42,48], we also examine the heterogeneous impact of inequality on property crime and violent crime. Violent crime is the sum total of the incidence of murder, attempt to commit murder, culpable homicide not amounting to murder and rape. Minor property crime is the sum total of the incidence of forgery, counterfeiting, and criminal breach of trust. Property crime is the sum total of the incidence of hurt, theft, criminal trespass burglary, dacoity, preparation and assembly for committing dacoity and arson. Hence, we report the results for the system GMM estimation based on equation (3) for violent crime, minor property crime and property crime rate across cities in India. However, since the National Crime Records Bureau (NCRB) does not provide the conviction rate for the subcategories across cities in India, we use the conviction rate for total crime as the explanatory variable in Table 3. The conviction rate for total crime would then capture the perception of the probability of conviction across cities in India.

**Table 3:** Impact of inequality on violent, minor property and property crime across Indian cities.

Dependent variable	Violent Crime	Minor property crime	Property crime
	(1)	(2)	(3)
	SystemGMM	SystemGMM	SystemGMM
L.log (crime rate)	0.640^***^	0.0354	0.372^***^
	(0.0723)	(0.124)	(0.126)
log (Nighttime light inequality)	0.500^***^	0.458^*^	0.466^+^
	(0.177)	(0.271)	(0.293)
log (conviction rate)	−0.0131	−0.0350	−0.0200
	(0.0243)	(0.0483)	(0.0179)
log (nighttime lights)	0.491	−1.387	−0.229
	(0.772)	(1.787)	(1.046)
log (nighttime lights)^2	−0.0238	0.128	0.0245
	(0.0421)	(0.110)	(0.0568)
log (population density)	−0.0717	−0.631^**^	−0.163^+^
	(0.0502)	(0.272)	(0.101)
Constant	−0.822	7.635	4.440
	(3.598)	(7.423)	(4.560)
Observations	242	241	242
No: of City	49	49	49
City fixed effect	Yes	Yes	Yes
Year fixed effect	Yes	Yes	Yes
Number of instruments	24	24	24
AR (1) test (p-value)	0.0187	0.0343	0.0066
AR (2) test (p-value)	0.1692	0.957	0.5951
Sargan test (p-value)	0.3622	0.4649	0.1982

L.log (crime rate) is the crime rate for violent crime, minor property crime and property crime lagged by one year. Crime rate is defined as the respective crime recorded per 100,000 persons. Inequality based on nighttime lights is the Gini coefficient of nighttime lights used as a proxy measure of inequality. All variables in the analysis have been log-transformed. Statistical significance is denoted as follows:

*** ,

** ,

* ,

+  statistically significant at the 1% level, 5% level, 10% level, and 12% level respectively. The p-values for the AR (1), AR (2), and Sargan overidentification tests are also reported.

Column (1), Column (2), and Column (3) in Table 3 provide the results for the system generalized method of moments estimation for violent crime, minor property crime and property crime rate, respectively. Column (1), Column (2) and Column (3) in Table 3 shows that a 1% increase in inequality based on nighttime lights leads to 0.5% increase in violent crime rate, 0.46% increase for both minor property crime rate and property crime rate, respectively. Although the sign for the conviction rate is negative, we do not find the conviction rate for total crime to have a statistically significant impact on violent crime, minor property crime and property crime across cities in India. Further, the coefficient for nighttime light inequality is positive and is statistically significant for violent crime after controlling for the impact of COVID-19 pandemic using dummy variable, and is provided in S1 Table 4 in S1 Appendix.

## 4. Discussion

Our study uses nighttime lights and its inequality as an alternative indicator for economic activity and economic inequality, respectively, due to the lack of socioeconomic indicator at the city level in India. Although several studies have shown inequality to have a positive effect on the incidence of crime [5,9,41,44,49], most studies examining crime at the subnational level or regional level in India omits inequality in the regression specification [12,40,42] due to the unavailability of inequality data [40]. The evidence from recent studies shows that inequality based on nighttime lights obtained from VIIRS satellites can be used as an alternative indicator for economic inequality, especially in developing countries with limited data on inequality [24,26,50,51]. In this context, our study contributes to the literature on nighttime light inequality as an alternative indicator for economic inequality to examine socio-economic outcomes, i.e., incidences of conflict in a country [52], electric power consumption inequality [53]. Our study examines the impact of inequality on crime rate for 49 Indian cities and finds evidence on the positive effect of nighttime light inequality on the total crime rate. Further, the positive impact of inequality on crime remains intact for violent crime and property crime with relatively higher coefficient of inequality for violent crime. However, the result is robust only for violent crime with the inclusion of Covid-19 dummy as an additional covariate (see S1 Table 4 in S1 Appendix). The differences in the statistical significance of the coefficient arises due to the variation in spread of the residuals across the regressions for different types of crime. Therefore, a ‘one size fits all’ regression approach can mask the heterogeneity in the determinants of crime across different crime types [54]. Additionally, because of the limitation in the availability of conviction rate for specific types of crime, we use the conviction rate for total crime as the legal deterrent factor [12]. Hence, the coefficient for conviction rate, although negative, are statistically significant only for total crime but not for violent, property or minor property crime.

One of the limitations of nighttime light inequality is its inability to fully capture the inequality across households in densely populated areas, i.e., a 1 km × 1 km spatial polygon for which nighttime lights is extracted may comprise of several rich and poor households [55]. Consequently, the diversity in the consumption or wealth across households in densely populated regions may not be fully reflected in nighttime light inequality. Therefore, complementing nighttime lights data by utilising alternative data sources such as mobile phone data, built up environment with machine learning models can provide more accurate measurement of economic inequality [56].

## 5. Conclusion

We study the impact of inequality on crime rate across 49 cities in India from 2016 to 2021. Most studies examining crime in India analyse the crime rate at the state and district level [12,40,42,49] due to the limited socioeconomic data at the city level. Therefore, limited quantitative studies on the detrimental effect of inequality on crime are available with cities as the unit of analysis in India. The novelty in our study is to use inequality based on nighttime lights obtained from VIIRS satellites as an alternative indicator for economic inequality across 49 Indian cities. Since indicators for economic inequality are limited and unavailable for Indian cities, inequality based on nighttime lights can be used as an alternative indicator for economic inequality at the city level [26,57].

Our findings suggest that inequality has a positive impact on total crime, violent crime, minor property crime and property crime across cities in India. Additionally, we also find that a higher probability of conviction lowers total crime rate across Indian cities. However, due to the limited availability of data on conviction rates for different crime types, we are unable to validate the same for different crime types. India, being a rapidly urbanizing and developing country, therefore needs to focus on uniform development within cities to reduce crime rates across cities.

## Supporting information

S1 Appendix Appendix tables. (DOCX)

S1 Data Replication data. (ZIP)
