# Supplementary material for: Economic inequality and crime across cities in India: Evidence using nighttime lights data
Source: PLoS One. 2025 Aug 11;20(8):e0324937. doi: 10.1371/journal.pone.0324937 (PMC12338796; doi:10.1371/journal.pone.0324937)
Supplement: S1 Appendix — (DOCX) [file pone.0324937.s001.docx]

**S1 Appendix**

**S1 Table 1**: Summary Statistics

| Variables | count | mean | sd | min | max |
| --- | --- | --- | --- | --- | --- |
| log(total crime rate) | 294 | 5.98 | 0.59 | 4.33 | 7.5 |
| log(violent crime rate) | 294 | 2.16 | 0.69 | 0.04 | 3.67 |
| log(minor property crime rate) | 293 | 1.19 | 1.16 | -1.63 | 4.17 |
| log(property crime rate) | 294 | 4.78 | 0.84 | 1.91 | 7.14 |
| log(nighttime light inequality) | 294 | -0.95 | 0.21 | -1.71 | -0.46 |
| log(conviction rate) | 291 | -5.92 | 1.46 | -10.81 | -3.28 |
| log(nighttime light) | 294 | 9.86 | 1.1 | 5.81 | 12.3 |
| log(nighttime light squared) | 294 | 98.34 | 20.98 | 33.71 | 151.32 |
| log(population density) | 294 | 8.06 | 1.07 | 5.82 | 10.6 |

**S1 Table 2**: Effect of inequality on Total Crime rate across Cities in India assuming linear relationship between crime rate and economic activity.

|  | Dependent variable: log(crime rate) | | |  |
| --- | --- | --- | --- | --- |
|  | (1) | (2) | (3) |  |
|  | FE | FE with lag | SystemGMM |  |
|  |  |  |  |  |
| L.log(crime rate) |  | 0.125 | 0.329** |  |
|  |  | (0.0905) | (0.151) |  |
| log(Nighttime light inequality) | 0.699** | 0.744* | 0.857** |  |
|  | (0.271) | (0.396) | (0.434) |  |
|  |  |  |  |  |
| log(conviction rate) | -0.0735* | -0.101** | -0.124* |  |
|  | (0.0370) | (0.0409) | (0.0690) |  |
|  |  |  |  |  |
| log(nighttime light) | -0.113 | 0.108 | 0.223* |  |
|  | (0.125) | (0.176) | (0.124) |  |
|  |  |  |  |  |
| log(population density) | 0.397 | 0.194 | -0.322* |  |
|  | (0.280) | (0.277) | (0.189) |  |
|  |  |  |  |  |
| Constant | 4.149 | 2.713 | 4.482*** |  |
|  | (2.485) | (2.789) | (1.272) |  |
|  |  |  |  |  |
| Observations | 291 | 242 | 242 |  |
| No: of City | 49 | 49 | 49 |  |
| City FE | Yes | Yes | Yes |  |
| Year FE | Yes | Yes | Yes |  |
| Within R squared | 0.124 | 0.142 |  |  |
| Number of instruments |  |  | 42 |  |
| AR1 test (p-value) |  |  | 0.0015 |  |
| AR2 test (p-value) |  |  | 0.5616 |  |
| Sargan test (p-value) |  |  | 0.4958 |  |
| L.log(crime rate) is the crime rate lagged by one year. Crime rate is defined as the total crime recorded per 100,000 population. Nighttime light inequality is the Gini coefficient of nighttime light used as a proxy measure of inequality. All variables in the analysis have been log-transformed. Statistical significance is denoted as follows: *** at the 1% level, ** at the 5% level, and * at the 10% level. Standard errors clustered at the city level are provided in parenthesis. The p-values for the AR(1), AR(2), and Sargan overidentification tests are also reported. | | | |  |
|  |  |  |  |  |

**S1 Table 3**: Effect of inequality on Total Crime rate across Cities in India assuming linear relationship between crime rate and economic activity with COVID-19 dummy.

|  | Dependent variable: log(crime rate) | | |  |
| --- | --- | --- | --- | --- |
|  | (1) | (2) | (3) |  |
|  | FE | FE with lag | SystemGMM |  |
|  |  |  |  |  |
| L.log(crime rate) |  | 0.114 | 0.313* |  |
|  |  | (0.0958) | (0.183) |  |
| log(Nighttime light inequality) | 0.660** | 0.702* | 0.700* |  |
|  | (0.264) | (0.386) | (0.387) |  |
|  |  |  |  |  |
| log(conviction rate) | -0.0807** | -0.110** | -0.163* |  |
|  | (0.0380) | (0.0427) | (0.0869) |  |
|  |  |  |  |  |
| log(nighttime light) | -0.136 | 0.0843 | 0.219 |  |
|  | (0.126) | (0.164) | (0.135) |  |
|  |  |  |  |  |
| log(population density) | 0.370 | 0.180 | -0.260 |  |
|  | (0.260) | (0.263) | (0.191) |  |
|  |  |  |  |  |
| Covid-19 dummy | 0.00598 | -0.0205 | -0.0273 |  |
|  | (0.0542) | (0.0589) | (0.0730) |  |
|  |  |  |  |  |
| Constant | 4.492* | 3.046 | 3.719*** |  |
|  | (2.240) | (2.532) | (1.273) |  |
|  |  |  |  |  |
| Observations | 291 | 242 | 242 |  |
| No: of City | 49 | 49 | 49 |  |
| City FE | Yes | Yes | Yes |  |
| Within R squared | 0.118 | 0.135 |  |  |
| Number of instruments |  |  | 39 |  |
| AR1 test (p-value) |  |  | 0.0102 |  |
| AR2 test (p-value) |  |  | 0.6208 |  |
| Sargan test (p-value) |  |  | 0.3632 |  |
| L.log(crime rate) is the crime rate lagged by one year. Crime rate is defined as the total crime recorded per 100,000 population. Nighttime light inequality is the Gini coefficient of nighttime light used as a proxy measure of inequality. All variables in the analysis have been log-transformed. Statistical significance is denoted as follows: *** at the 1% level, ** at the 5% level, and * at the 10% level. Standard errors clustered at the city level are provided in parenthesis. The p-values for the AR(1), AR(2), and Sargan overidentification tests are also reported. Covid-19 dummy for the year 2020 and 2021 are marked as 1, otherwise 0. | | | |  |
|  |  |  |  |  |

**S1 Table 4**: Effect of inequality on inequality on Violent crime, Minor Property crime and Property crime across Cities in India with COVID-19 dummy.

| Dependent variable | Violent Crime | Minor property crime | Property crime |  |
| --- | --- | --- | --- | --- |
|  | (1) | (2) | (3) |  |
|  | SystemGMM | SystemGMM | SystemGMM |  |
|  |  |  |  |  |
| L.log(crime rate) | 0.494*** | -0.0597 | 0.0236 |  |
|  | (0.154) | (0.181) | (0.0474) |  |
|  |  |  |  |  |
| log(Nighttime light inequality) | 0.522+ | 0.728 | 0.0710 |  |
|  | (0.324) | (0.525) | (0.254) |  |
|  |  |  |  |  |
| log(conviction rate) | -0.00525 | 0.0228 | 0.0107 |  |
|  | (0.0256) | (0.0568) | (0.0145) |  |
|  |  |  |  |  |
| log(nighttime light) | 0.121 | -2.804 | 0.872 |  |
|  | (1.648) | (4.762) | (1.386) |  |
|  |  |  |  |  |
| log(nighttime light)^2 | 0.00110 | 0.205 | -0.0221 |  |
|  | (0.0905) | (0.262) | (0.0753) |  |
|  |  |  |  |  |
| log(population density) | -0.158 | -0.718 | -0.393*** |  |
|  | (0.110) | (0.471) | (0.102) |  |
|  |  |  |  |  |
| Covid-19 dummy | -0.0427 | -0.191** | -0.0818*** |  |
|  | (0.0523) | (0.0921) | (0.0296) |  |
|  |  |  |  |  |
| Constant | 1.545 | 15.33 | 1.650 |  |
|  | (7.715) | (22.78) | (6.301) |  |
|  |  |  |  |  |
| Observations | 242 | 241 | 242 |  |
| No: of City | 49 | 49 | 49 |  |
| City FE | Yes | Yes | Yes |  |
| Number of instruments | 21 | 21 | 21 |  |
| AR1 test (p-value) | 0.0274 | 0.1186 | 0.0597 |  |
| AR2 test (p-value) | 0.0938 | 0.7578 | 0.6539 |  |
| Sargan test (p-value) | 0.0475 | 0.2707 | 0.1643 |  |
| L.log(crime rate) is the crime rate for violent crime, minor property crime and property crime lagged by one year. Crime rate is defined as the crime recorded per 100,000 population. Nighttime light inequality is the Gini coefficient of nighttime light used as a proxy measure of inequality. All variables in the analysis have been log-transformed. Statistical significance is denoted as follows: *** at the 1% level, ** at the 5% level, * at the 10% level, and + at the 12% level. The p-values for the AR(1), AR(2), and Sargan overidentification tests are also reported. Covid-19 dummy for the year 2020 and 2021 are marked as 1, otherwise 0. | | | |  |
|  |  |  |  |  |
